# Bugs and Fleas

**Published:** 1879-08

**Authors:** 


### BUGS AND FLEAS.

Bed-bugs are readily exterminated by wetting the joints or the ends of the slats of bedsteads with a mixture of Paris-green and kerosene oil; a teaspoonful of the Paris-green to a pint of oil. But fleas in some countries are an intolerable nuisance, and give more annoyance than all other insects combined; and it is impossible to get rid of them. A few grains of gum-camphor, made fine and sprinkled between the sheets just before going to bed, will prevent their raids. A few drops of carbolic acid diluted in a little water and sprinkled on the sheets is still better.

